# Online Cognitive Testing for the Assessment of Alzheimer's Disease Risk and Early Disease Stages

**DOI:** 10.1002/alz70856_099508

**Published:** 2025-12-24

**Authors:** Lina Gomez, Jonny P Flint, Jessica Adsett, Kerrie McAloney, Renate Thienel, Léonie Borne, Caroline Faucher, Gail Robinson, Nicholas G Martin, Michael J Breakspear, Michelle K Lupton

**Affiliations:** ^1^ QIMR Berghofer Medical Research Institute, Brisbane, QLD, Australia; ^2^ The University of Newcastle, Callaghan, NSW, Australia; ^3^ The University of Queensland, Brisbane, QLD, Australia

## Abstract

**Background:**

Self‐administered online cognitive testing offers a cost‐effective and flexible alternative to in‐person neuropsychological assessments. We used the Creyos platform (previously named Cambridge Brain Sciences) to assess cognitive function (including short‐term memory, reasoning, verbal ability, and concentration) in middle aged and older adults (aged 42‐75) participating in PISA (the Prospective Imaging Study of Aging). PISA is one of the largest cohort studies in the world to focus on the identification of prodromal and early‐stage biomarkers for Alzheimer's disease.

**Methods:**

We validated the Creyos platform in 141 healthy PISA participants by comparing the results with a comprehensive in‐person cognitive assessment. We used a multivariate technique to study the ability of each test to inform brain morphology in the form of cortical sulcal width extracted from structural magnetic resonance imaging.

Utilising our large sample (*N* = 2,000 baseline and N>1,000 follow‐up time points administered over one year later), we then investigated the association of Creyos cognitive measures with risk factors and early‐stage biomarkers for Alzheimer's disease.

**Results:**

The online tests demonstrated comparable strength of association with cortical sulcal width compared to the comprehensive in‐person assessment. We tested for an association of cognitive test scores with genetic risk for Alzheimer's disease. We identified a nominal association of Alzheimer's disease Polygenic Risk Scores (PRS) with reduced performance on four out of twelve of the cognitive tests covering executive function, memory, and visuospatial domains. Further work will be presented investigating the association of online cognitive testing results (including baseline measures and cognitive decline) with Alzheimer's disease risk factors and early‐stage biomarkers.

**Conclusions:**

Our findings suggest that, in our at‐risk sample, online assessments are comparable to in‐person neuropsychological testing in their association with brain morphology. Our results highlight the utility of online cognitive testing as a cost‐effective alternative to in‐person testing for large‐scale studies and the potential for it to be used as a pre‐screening tool to identify those at high risk or in the earliest disease stages of Alzheimer's disease. Online cognitive testing could lead to more equitable early detection and intervention for neurodegenerative diseases.

